# Modification of the drug resistance of emerging milk-borne pathogens through sodium alginate-based antibiotics and nanoparticles

**DOI:** 10.3389/fvets.2023.1130130

**Published:** 2023-04-17

**Authors:** Abdul Manan, Amjad Islam Aqib, Ansa Shahbaz, Shanza Rauf Khan, Kashif Akram, Hamid Majeed, Afshan Muneer, Maheen Murtaza, Muhammad Afrasiab, Carmine Merola, Kamal Niaz, Irfan Ahmad, Mohd Saeed

**Affiliations:** ^1^Department of Food Science, Cholistan University of Veterinary and Animal Sciences, Bahawalpur, Pakistan; ^2^Department of Medicine, Cholistan University of Veterinary and Animal Sciences, Bahawalpur, Pakistan; ^3^Basic Health Unit, Health Department Punjab, Tehsil Tandlianwala, Faisalabad, Pakistan; ^4^Department of Chemistry, University of Agriculture, Faisalabad, Pakistan; ^5^Department of Zoology, Cholistan University of Veterinary and Animal Sciences, Bahawalpur, Pakistan; ^6^Faculty of Bioscience and Technology for Food, Agriculture and Environment, University of Teramo, Teramo, Italy; ^7^Department of Pharmacology and Toxicology, Faculty of Bio-Sciences, Cholistan University of Veterinary and Animal Sciences, Bahawalpur, Pakistan; ^8^Department of Clinical Laboratory Sciences, College of Applied Medical Sciences, King Khalid University, Abha, Saudi Arabia; ^9^Department of Biology, College of Sciences, University of Hail, Hail, Saudi Arabia

**Keywords:** *Klebsiella pneumoniae*, *Streptococcus agalactiae*, dairy milk, antibiotic resistance, MgO nanoparticles, sodium alginate, tylosin, ampicillin

## Abstract

*Streptococcus agalactiae* and *Klebsiella pneumoniae* are emerging as major milk-borne pathogens. Additionally, resistance to antibiotics of pathogens is of concern. Therefore, this study investigated the prevalence and drug resistance of *S. agalactiae* and *K. pneumoniae* in mastitis milk samples and assessed the antimicrobial potential of sodium alginate (G)-stabilized MgO nanoparticles (M) and antibiotics (tylosin [T] and ampicillin [A]) against both of these pathogens. A total of *n* = 200 milk samples from cattle were collected using purposive sampling, and standard microbiological approaches were adopted to isolate target bacteria. Parametric and non-parametric statistical tests were used to analyze the obtained data. Four preparations, GT (gel-stabilized tylosin), GA (gel-stabilized ampicillin), GTM (tylosin and MgO nanoparticles stabilized in gel), and GAM (ampicillin and MgO nanoparticles stabilized in gel), were evaluated against both bacteria through well diffusion and broth microdilution method. The analysis revealed that 45.24% (95/210) of the milk samples were positive for mastitis, of which 11.58% (11/95) were positive for *S. agalactiae* and 9.47% (9/95) were positive for *K. pneumoniae*. *S. agalactiae* had a significantly higher zone of inhibition (ZOI) than *K. pneumoniae* against penicillin, tetracycline, and amoxicillin, whereas the opposite was observed against imipenem and erythromycin. All gel (G)-based preparations showed an increase in the percentage of ZOI compared with antibiotics alone, with GTM presenting the highest of all, i.e., 59.09 and 56.25% ZOI compared with tylosin alone against *S. agalactiae* and *K. pneumoniae*, respectively. Similarly, in a broth microdilution assay, the lowest MIC was found for *K. pneumoniae* (9.766 ± 0.0 μg/mL) against GTM, followed by GT, GAM, and GA after incubation for 24 h. A similar response was noted for preparations against *S. agalactiae* but with a comparatively higher MIC. A significant reduction in MIC with respect to incubation time was found at 8 h and remained until at 20 h against both pathogens. The cytotoxicity of the MgO nanoparticles used in this study was significantly lower than that of the positive control. Overall, this study found that *K. pneumoniae* and *S. agalactiae* appeared higher in prevalence and antimicrobial resistance, and sodium alginate-based antibiotics and MgO nanoparticles were effective alternative approaches for tackling antimicrobial resistance.

## 1. Introduction

The gap between milk production and supply is increasing despite the fact that Pakistan is one of the top milk-producing countries. The average shortfall in Karachi, a major cosmopolitan city, has already been estimated to reach 4 million liters per day. Milk consumption is expected to increase by a minimum of 5% annually in the future (1). Additionally, it is notable that an increase in milk yield is hampered by the bacterial attack on the udder of animals, which in turn, compromises animal and public health. There are several bacteria involved, salient of which are *Staphylococcus aureus, Escherichia coli, Corynebacterium* spp., *Streptococcus* spp., and *Klebsiella* spp. In recent years, minor species of bacteria have increased because of drug resistance. *Klebsiella pneumoniae* has been designated as a “critical” bacterium for the research and development of novel antibiotics due to its weak antibacterial susceptibility (2). *Streptococcus agalactiae*, now known as group B *Streptococcus* (GBS), was differentiated from other *Streptococci* in the 1930s after it was identified in milk and cows suffering from bovine mastitis. Additionally, this pathogen is an intracellular parasite of the cow mammary gland and is highly infectious. Both of these pathogens have recently been reported to be increasing in dairy cattle in China and Egypt (3, 4). Furthermore, the development of antiobiotic resistance is an inevitable phenomenon. Recently, studies have reported considerable variations in the efficacy of antibiotics against both pathogens, clearly indicating their increasing resistance (5).

There is a dire need to counter antimicrobial resistance, for which reduction, refinement, replacement, review, and responsible use of antibiotics has been the recent approach. Nanoparticles are emerging as an effective replacement for antimicrobials against a wide range of pathogens. The use of nanoparticles in biomedicine has not only been applied for antimicrobial replacement (6, 7) but also for the improvement of meat quality (8) and has a significant role in antioxidant enzymes (9). A wide range of metallic and non-metallic nanoparticles are being applied in various products and other applications have also being evaluated in recent studies. Metallic MgO nanoparticles play a significant role in biological and applied sciences. Additionally, it is important to use antibiotics by coupling with a non-antibiotic source, such as chitosan or sodium alginate, to enhance efficacy at reduced quantity. Sodium alginate is a natural and hydrophilic anionic polysaccharide extracted from brown marine algae (*Phaeophyceae*), and it has been extensively applied in the food, bioengineering, and pharmaceutical fields. The inclusion of alginate in pectin-based formulations improves the strength of the zinc ions crosslinking network, which means it increases the capacity of the nanoparticle (10). The use of alginates has been proven to be safe, has excellent biocompatibility and biodegradability, and is highly effective at thickening and gelling products. They are hypothesized to enhance the antimicrobial activity of antimicrobial candidates. Recently, sodium alginate has been successfully used as a reducer and stabilizer of metal nanoparticles at different reaction times and temperatures in studies to examine other potential applications. Sodium alginate-stabilized silver nanoparticles have served as an effective antibacterial composite against gram-positive and gram-negative pathogens and have thus increased the shelf life of food (11, 12).

The bacteria selected in this study have commonly been overlooked for their prevalence and emerging resistance to drugs which is now being otherwise. On the other hand, the application of composite therapy consisting of conventional and non-conventional potential antimicrobial candidates is highly desirable. With this scenario in mind, the current study planned to estimate the prevalence of *K. pneumoniae* and *S. agalactiae* in mastitis milk samples, assessing their response to antibiotics, and examining their resistance modulation potential through sodium alginate-stabilized antibiotics and nanoparticles.

## 2. Results

### 2.1. Prevalence and antimicrobial susceptibility profile

**Figure 1:**
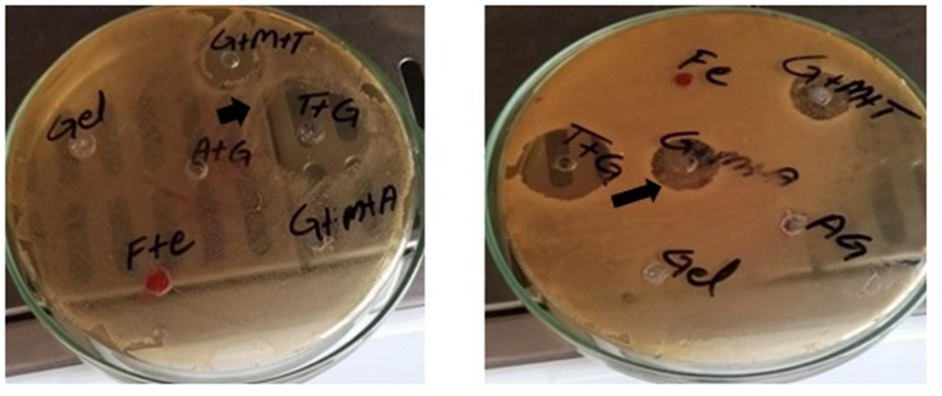
Zone of inhibitions (black arrows) for different preparations against *K. pneumoniae* **(left)** and *S. agalactiae* **(right)**.

This study revealed that 45.24% (95/210) of the dairy cattle milk samples were positive for mastitis. Of these mastitis samples, 11.58% (11/95) were positive for *S. agalactiae* and 9.47% (9/95) were positive for *K. pneumoniae*. Regarding the resistance of these bacteria, there was variation in the responses of *K. pneumoniae* and *S. agalactiae* against antibiotics in general (Table 1). For penicillin, tetracycline, and amoxicillin, *S. agalactiae* had considerably higher zones than *K. pneumoniae*. On the other hand, *K. pneumoniae* showed significant differences in zones of inhibition to those of *S. agalactiae* against imipenem and erythromycin (Figure 1). There were minor variations in antibacterial responses between both bacteria against ceftriaxone. Standard deviation from the mean value of the ZOI was higher for *K. pneumoniae* against cetirizine (6.14 mm) and imipenem (5.12 mm) than *S. agalactiae* against ceftriaxone (4.97 mm), penicillin (3.56 mm), and erythromycin (3.24 mm). This reflects the fact that the latter pathogen is much more inclined toward a single susceptibility category, whereas the former exhibits variable responses against antibiotics. The response of *S. agalactiae* (2.75 ± 0.83 mm) was nearly 10 times lower than that of *K. pneumoniae* (21.5 ± 2.96 mm) against aztronam.

**Table 1:** Zone of inhibitions (mm) shown by *K. pneumoniae* and *S. agalactiae* against antibiotics.

**Name of antibiotic**	**Abbreviation**	***K. pneumoniae***	***S. agalactiae***
		**Mean** ±**SD**	**Mean** ±**SD**
Imipenem	IMI	29.75 ± 5.12	19 ± 1.73
Penicillin	P10	19 ± 3.00	27.75 ± 3.56
Tetracycline	TE	20.75 ± 2.38	26.25 ± 1.09
Ampicillin	AMP	21 ± 2.12	28.25 ± 1.48
Ceftriaxone	CRO	22 ± 3.54	22.25 ± 4.97
Amoxicillin	AUG	20.5 ± 0.87	27 ± 2.12
Cefepime	FEP	20.25 ± 1.64	26 ± 2.92
Aztronam	ATM	21.5 ± 2.96	2.75 ± 0.83
Erythromycin	ERY	26.25 ± 2.86	23 ± 3.24

Zones of inhibitions are measured in millimeters (mm). SD, standard deviation.

### 2.2. Characterization of nanoparticles

**Figure 2:**
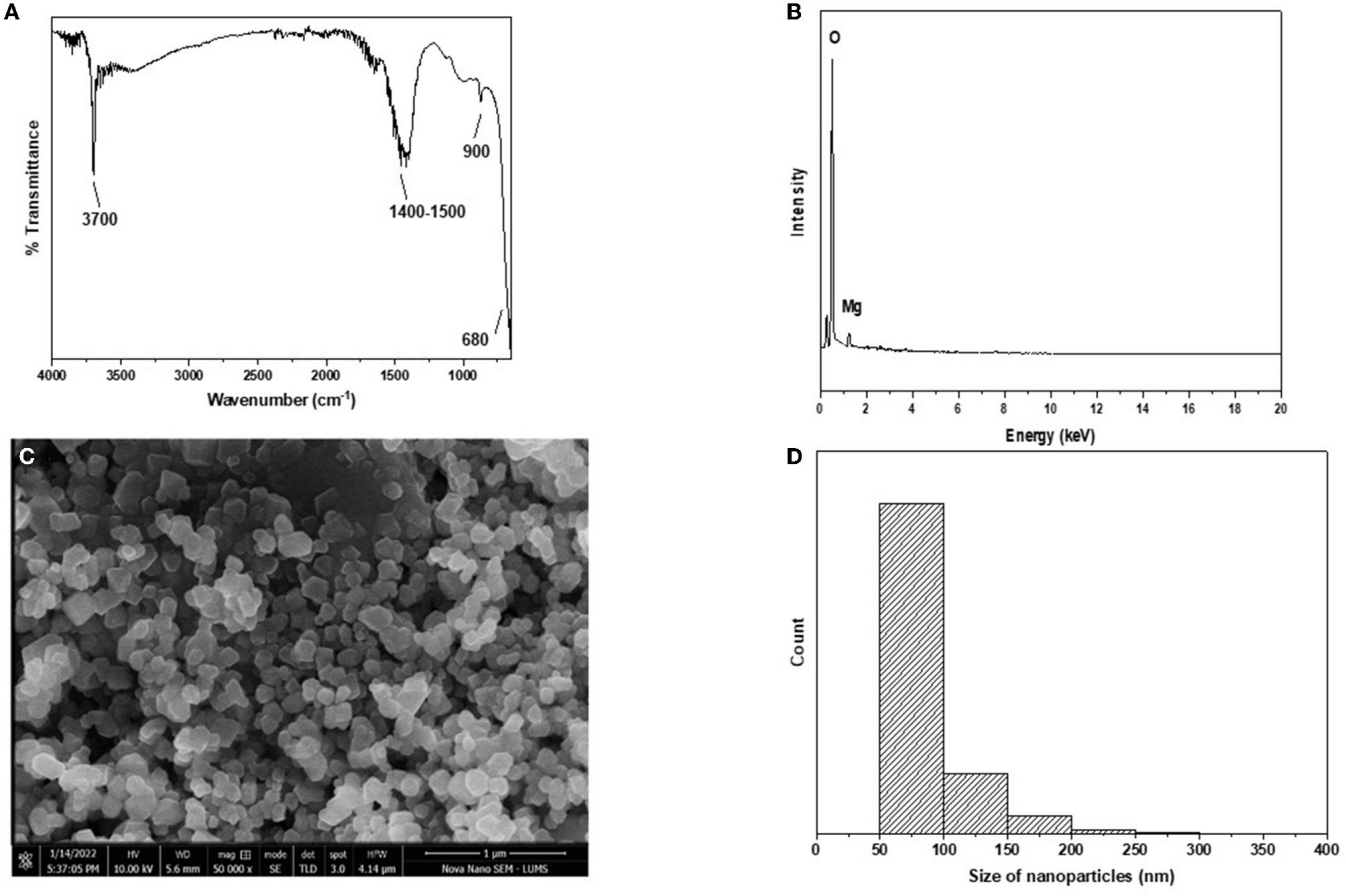
**(A–D)** Characterization of MgO nanoparticles. **(A)** FTIR. **(B)** EDX. **(C)** STEM. **(D)** Size distribution histogram.

The FTIR pattern of nanoparticles is shown in Figure 2A. The characteristic peak of the Mg-O bond was observed at a wavelength of 680 nm. This confirms that the metal-oxygen bond was present in the nanoparticles. Surfactant sodium dodecyl sulfate is an organic molecule and contains a C-H bond. Its band was observed at ~1,400–1,500 cm^−1^ due to the adsorption of surfactant on the surface of the nanoparticles. Water molecules present in the atmosphere are quickly adsorbed on the surface of the nanoparticles as soon as they are exposed to the atmosphere. The band at ~3,700 cm^−1^ shows the presence of an O-H bond due to water molecules.

EDX analysis of nanoparticles is shown in Figure 2B. Characteristic peaks of magnesium and oxygen are present in the plot. This confirms that magnesium and oxygen are present in the nanoparticles, which are MgO. Carbon tape was used for sample handling; hence, a peak of carbon can be observed.

STEM analysis of the nanoparticles is shown in Figure 2C. Spherical or oval-shaped nanoparticles were formed, ranging from 50 to 300 nm in size. The surface of the nanoparticles appeared to be smooth. The size distribution plot was drawn using data from STEM and shows that most of the nanoparticles ranged from 50 to 150 nm in size (Figure 2D), although a few nanoparticles were larger. Very small nanoparticles may have been attached to the surface of the other nanoparticles, thus making them larger than the others. XRD produced diffracted peaks for the nanoparticles that were narrow at higher temperatures. The Miller index at 2 theta angles 43.0°, 46.0°, 63.0°, 75.0°, and 78.0° were noted at 111, 200, 220, 311, and 222, respectively. These nanoparticles belonged to the face-centered cubic structure and Fm-3m space group, thus confirming the synthesis of MgO nanoparticles. SEM analysis showed a rounded cubical-shaped structure with corners that were not pointed but rather rounded or curved. The boundaries of these nanoparticles were smooth and clear. The size of the prepared nanoparticles ranged between 80 and 200 nm (approximately), and they had a well-dispersed shape rather than an aggregate form (Figure 2).

### 2.3. Toxicity analysis

The cytotoxicity study revealed a significant difference between MgO-treated groups and the controls (Table 2). DNA damage was significantly (*p* < 0.05) lower in the MgO-treated groups than in the positive control after 24 and 48 h of contact time. However, DNA damage was directly proportional to the concentration of MgO nanoparticles, with a significant difference between each other, except between concentrations of 2.5 mg/mL and 5 mg/mL after 48 h of contact time. On the other hand, the phase index increased with an increase in the concentration of MgO up to 2.5 mg/mL; the phase index decreased beyond this concentration. This finding thus suggests that MgO nanoparticles are safe as their toxicity is significantly lower than the positive control.

**Table 2:** Effect of magnesium oxide nanoparticles on the phase index and DNA damage in *A. cepa*.

**Concentration (mg/mL)**	**CCN**	**Phase index (%)** ±**SD**				**DNA damage**
		**Prophase**	**Metaphase**	**Anaphase**	**Telophase**	
**24 h of contact time**						
Control (negative control)	506	90.09 ± 0.420^a^	3.02 ± 0.11^a^	3.62 ± 0.28^a^	6.98 ± 0.19^a^	18.11 ± 0.12^e^
MMS (positive control)	507	84.130 ± 0.02^b^	1.01 ± 0.16^d^	1.99 ± 0.01^b^	3.88 ± 0.87^b^	135.0 ± 0.22^a^
1.25 mg/mL M	540	81.120 ± 0.240^c^	2.150 ± 0.12^b^	1.810 ± 1.260^b^	2.320 ± 0.22^c^	110.±0.10^d^
2.5 mg/mL M	550	80.22 ± 0.12^d^	2.09 ± 0.01^b^	1.800 ± 0.220^b^	2.13 ± 0.41^c^	112.±0.45^c^
5 mg/mL M	565	78.23 ± 0.110^e^	1.76 ± 0.11^c^	1.22 ± 0.19^b^	1.99 ± 0.31^c^	115.0 ± 0.35^b^
**48 h of contact time**						
Control	506	90.090 ± 0.420^a^	3.02 ± 0.11^a^	3.620 ± 0.280^a^	6.98 ± 0.190^a^	18.11 ± 0.12^d^
MMS	507	84.130 ± 0.020^b^	1.01 ± 0.16^c^	1.99 ± 0.01^ab^	3.88 ± 0.87^b^	135.0 ± 0.22^a^
1.25 mg/mL M	555	78.99 ± 0.55^c^	1.89 ± 0.03^b^	1.99 ± 1.46^ab^	2.65 ± 0.45^bc^	112.0 ± 0.11^c^
2.5 mg/mL M	507	83.12 ± 0.33^b^	1.69 ± 0.04^b^	1.86 ± 0.31^ab^	2.450 ± 0.50^c^	120.0 ± 0.550^b^
5 mg/mL M	540	80.03 ± 0.56^c^	1.66 ± 0.11^b^	1.77 ± 0.17^b^	1.53 ± 0.28^c^	120.0 ± 0.99^b^

Different superscript letters in the same column show a statistically significant difference (*p* < 0.05) at 24 and 48 h. MMS, methyl methane sulphonate; CCN, counting cell numbers; M, MgO nanoparticles. NB: The averages of the positive and negative controls were first assessed for the whole duration and set as the standard for the comparison of MgO concentrations after 24 and 48 h of contact time. SD, Standard deviation.

### 2.4. Resistance modulation through gel-stabilized preparations

#### 2.4.1. Comparison of composites with single antibiotics (well diffusion assay)

This study showed a significant difference in the ZOI (mm) between the sodium alginate-stabilized antibiotics and/or MgO nanoparticles compared with the antibiotics alone. GT and GTM were non-significantly different with each other in terms of antibacterial activity but were significantly different (*p* < 0.05) from tylosin alone (Table 3, Figure 1). A similar response was noted when gel-stabilized antibiotic and antibiotic plus MgO nanoparticles were compared with ampicillin (A) alone. There was a more than 50% increase in antibacterial activity in both gel-impregnated tylosin and tylosin plus MgO nanoparticles compared with T alone. On the other hand, GA and GAM showed a 47.28 and 53.112% increase in antibacterial activity compared with that of A alone. GT and GTM showed significantly higher responses compared with tylosin alone and an increase in the ZOI of 54.93 and 56.25%, respectively, against *S. agalactiae*. A comparison between ampicillin and GA and GAM revealed a significant difference (*p* < 0.05) among all in that the latter showed a significantly higher ZOI than A and GA. GA and GTM increased the ZOI by 43.91 and 53.80%, respectively.

**Table 3:** Comparison of the antibacterial activity (zone of inhibition) of sodium alginate-stabilized antibiotic and nanoparticles against bacteria.

**Antibiotic**	**Combinations**	***S. agalactiae***		***K. pneumoniae***	
		**Mean** ±**SD**	**% change**	**Mean** ±**SD**	**% Change**
**T**	Alone	12.47 ± 0.950^b^	–	13.80 ± 0.608^b^	–
	GT	27.67 ± 1.528^a^	54.93%	31.17 ± 2.75^a^	54.74%
	GTM	28.500 ± 1.000^a^	56.25%	33.73 ± 0.681^a^	59.09%
**A**	Alone	9.47 ± 1.002^c^	–	10.37 ± 0.850^b^	–
	GA	16.83 ± 0.764^a^	43.91%	19.67 ± 2.08^a^	47.28%
	GAM	20.50 ± 1.000^b^	53.80%	22.33 ± 1.258^a^	53.112%

The percentage change in the ZOI of gel-stabilized preparations was compared with the antibiotic alone. It was measured as (e.g., GT compared with T alone) = (GT-T/GT)^*^100. SD, Standard deviation.

Different superscript letters within column for T (tylosin) indicate significant difference (*p* < 0.05) for bacteria. Same is in the case of A (ampicillin).

#### 2.4.2. Comparison of composites with single antibiotics (broth microdilution assay)

This study indicated the efficacy of antibiotics at lower concentrations when stabilized in sodium alginate in combination with nanoparticles. The lowest MIC against *K. pneumoniae* was observed with GTM (9.766 ± 0.000 μg/mL), followed by GT, GAM, and GA after 24 h of incubation (Table 4). There were significant differences (*p* < 0.05) in MICs between GT and GAM or GTM, whereas GTM and GA were not significantly different (*p* > 0.05) from each other, as were GA and GT (*p* > 0.05). A similar response of alginate-stabilized products was observed against *S. agalactiae* (Table 5). However, the magnitude of MICs was relatively higher than those noted in the case of *K. pneumoniae*. The lowest MIC was observed with GTM (26.04 ± 11.28 μg/mL) followed by GT (39.06 ± 0.00 μg/mL), whereas GA and GAM had the same MIC (78.13 ± 0.00 μg/mL). The MIC of GT and GTM was significantly different to that of GAM and GA. However, GA and GAM were not significantly different (*p* > 0.05) from each other, as were GT and GTM (*p* > 0.05).

**Table 4:** Comparison of the minimum inhibitory concentrations (μg/mL) of different gel-based preparations against *K. pneumoniae* at each time interval.

**Preparations**	**4 h**	**8 h**	**12 h**	**16 h**	**20 h**	**24 h**
GT	625.0 ± 0^a^	260.4 ± 90.2^a^	130.2 ± 45.1^a^	78.13 ± 0.00^a^	52.1 ± 226^a^	19.53 ± 0.00^a^
GA	625.0 ± 0^a^	260.4 ± 90.2^a^	156.25 ± 0.0^a^	130.2 ± 45.1^a^	52.1 ± 22.6^a^	39.06 ± 0.00^ab^
GTM	417 ± 180^a^	156.25 ± 0.0^a^	78.13 ± 0.00^a^	65.1 ± 22.6^a^	19.53 ± 0.00^b^	9.766 ± 0.000^bc^
GAM	521 ± 10^a^	260.4 ± 90.2^a^	130.2 ± 45.1^a^	78.13 ± 0.00^a^	39.06 ± 0.00^c^	26.04 ± 11.28^c^

GT, gel-stabilized tylosin; GA, gel-stabilized ampicillin; GTM, tylosin and MgO nanoparticles stabilized in gel; GAM, ampicillin and MgO stabilized in gel.

Different superscript letters within a column indicate a significant difference (*p* < 0.05).

**Table 5:** Comparison of the minimum inhibitory concentrations (μg/mL) of different gel-based preparations against *S. agalactiae* at each time interval.

**Preparations**	**4 h**	**8 h**	**12 h**	**16 h**	**20 h**	**24 h**
GT	833 ± 361^a^	313 ± 271^a^	156.25 ± 0.0^a^	156.25 ± 0.0^a^	65.1 ± 22.6^ab^	39.06 ± 0.00^b^
GA	1042 ± 361^a^	625.0 ± 0.0^a^	469 ± 271^a^	130.2 ± 45.1^a^	130.2 ± 45.1^a^	78.13 ± 0.00^b^
GTM	521 ± 180^a^	260.4 ± 90.2^a^	208.3 ± 90.2^a^	130.2 ± 45.1^a^	39.06 ± 0.00^b^	26.04 ± 11.28^b^
GAM	625.0 ± 0.0^a^	312.5 ± 0.0^a^	208.3 ± 90.2^a^	130.2 ± 45.1^a^	104.2 ± 45.1^ab^	78.13 ± 0.00^a^

GT, gel-stabilized tylosin; GA, gel-stabilized ampicillin; GTM, tylosin and MgO nanoparticles stabilized in gel; GAM, ampicillin and MgO stabilized in gel.

Different superscript letters within a column indicate significant differences (*p* < 0.05).

#### 2.4.3. Effect of incubation interval on the activity of composite

**Figure 3:**
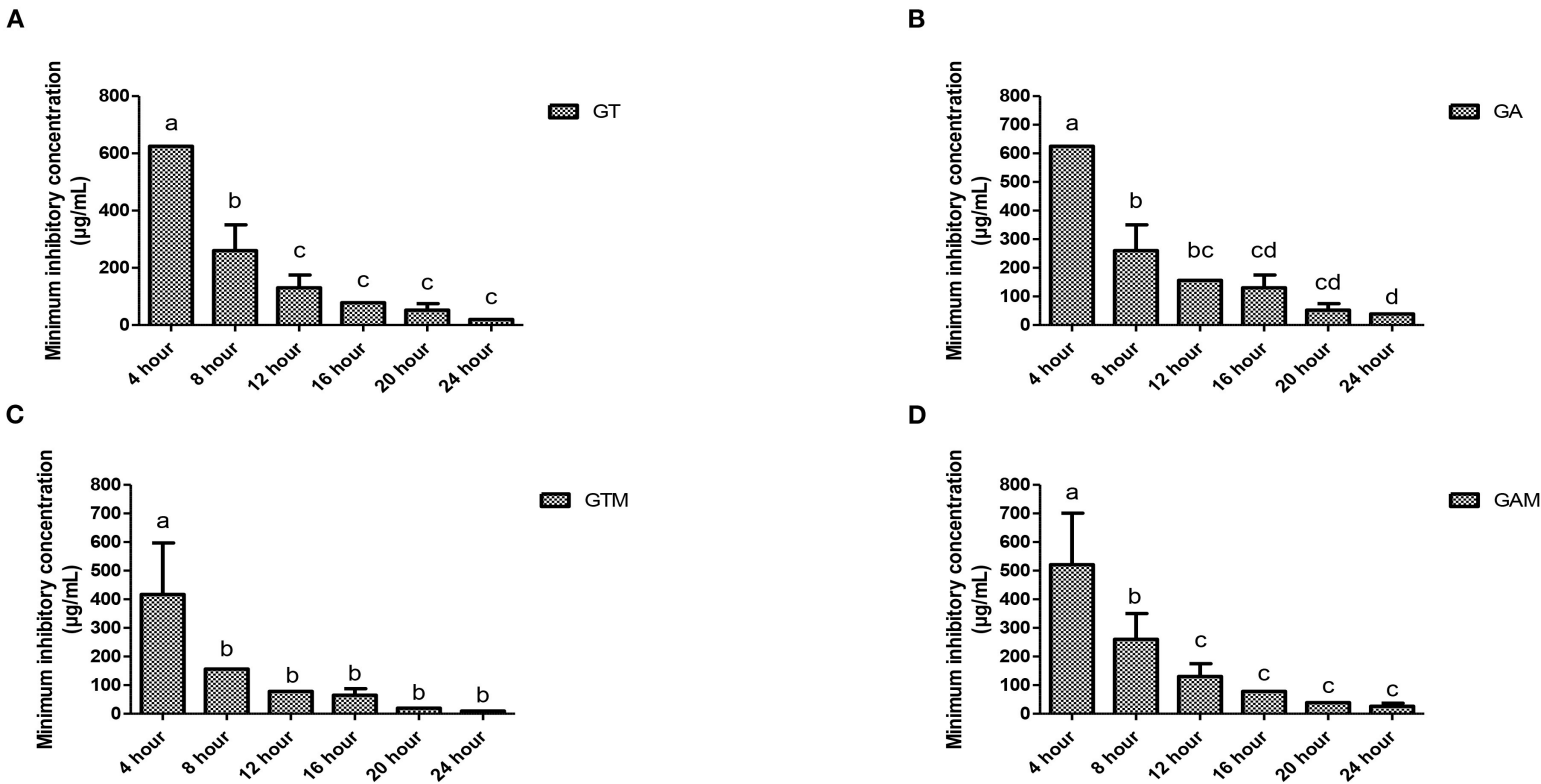
**(A–D)** Comparison of minimum inhibitory concentrations (μg/mL) among different incubation times for each preparation against *K. pneumoniae*. **(A)** GT, sodium alginate-stabilized tylosin. **(B)** GA, sodium alginate-stabilized ampicillin. **(C)** GTM, sodium alginate-stabilized tylosin and MgO nanoparticles. **(D)** GAM, sodium alginate-stabilized ampicillin and MgO nanoparticles.

**Figure 4:**
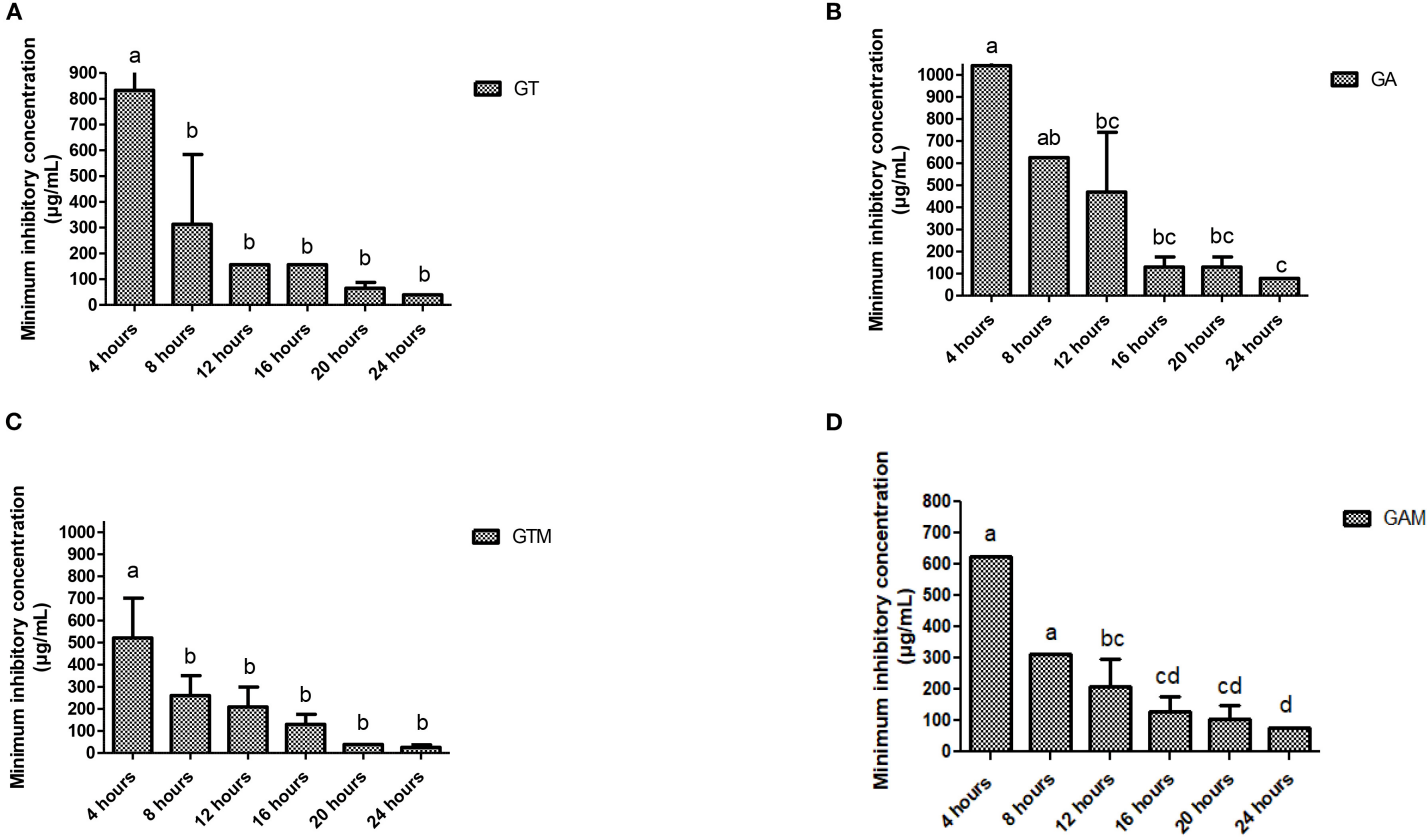
**(A–D)** Comparison of minimum inhibitory concentrations (μg/mL) among different incubation times for each preparation against *S. agalactiae*. **(A)** GT, sodium alginate-stabilized tylosin. **(B)** GA, sodium alginate-stabilized ampicillin. **(C)** GTM, sodium alginate-stabilized tylosin and MgO nanoparticles. **(D)** GAM, sodium alginate-stabilized ampicillin and MgO nanoparticles.

The efficacy of composite to respond at various time intervals of incubation differed significantly thus reflecting the availability of several effective dose to response time options. Each preparation at different time intervals showed a variable response (Figure 3). GT showed a significant reduction in MIC after 8 h of incubation that continued to differ until 12 h of incubation, indicating that GT can achieve the highest response after 12 h of incubation. GA showed variable means of MICs at different time intervals showing significant differences (*p* < 0.05) at initial hours and last hours while the middle hours of incubation remained non-significant (*p* > 0.05) different to each other. The same kind of response was observed for GAM, while GTM exhibited a comparatively smoother decline in MICs, showing a significant time-dependent response of MICs against *K. pneumoniae* (Figure 3). The antibacterial activity of GTM showed the lowest MIC (26.04 ± 11.28 μg/mL) followed by GT (39.06 ± 0.00 μg/mL), while GA and GAM showed the same MIC (78.13 ± 0.00 μg/mL) against *S. agalactiae* (Figure 4). The incubation period showed a significant reduction in MIC after 8 h of incubation for each preparation, which was sustained until 16 h of incubation. GTM and GT showed a uniform response of non-significant reduction in MIC after 8 h of incubation, indicating that effective antibacterial activity was obtained in a short incubation period.

### 2.5. Comparision of minimum inhibitory concentration (μg/mL) of *K. pneumoniae* and *S. agalactiae* against different gel-based preparations

The mean and standard deviation of the comparison of the MIC between *K. pneumoniae* and *S. agalactiae* showed a non-significant difference. GT, GA, GTM, and GAM had means and standard deviations (μg/mL) of 39.06 ± 0.00 and 19.53 ± 0.00; 78.13 ± 0.00 and 39.06 ± 0.00; 26.04 ± 11.28 and 9.766 ± 0.000; and 78.13 ± 0.00 and 26.04 ± 11.28, respectively, at 24 h against *K. pneumoniae* and *S. agalactiae*, respectively (Table 6).

**Table 6:** Minimum inhibitory concentration (μg/mL) of *K. pneumoniae* and *S. agalactiae* against different gel-based preparations.

**Nanoparticle and antibiotic**	**Bacteria**	**4 h**	**8 h**	**12 h**	**16 h**	**20 h**	**24 h**
GT	*Streptococcus*	625.0 ± 0.0^a^	260.4 ± 90.2^a^	130.2 ± 45.1^a^	78.13 ± 0.00^a^	52.1 ± 22.6^a^	19.53 ± 0.00^a^
	*Klebsiella*	833 ± 361^a^	313 ± 271^a^	156.25 ± 0.0^a^	156.25 ± 0.0^a^	65.1 ± 22.6^a^	39.06 ± 0.00^a^
GA	*Streptococcus*	625.0 ± 0.0^a^	260.4 ± 90.2^a^	156.3 ± 0.0^a^	130.2 ± 45.1^a^	52.1 ± 22.6^a^	39.06 ± 0.00^a^
	*Klebsiellae*	1042 ± 361^a^	625.0 ± 0.0^b^	469 ± 271^a^	130.2 ± 45.1^a^	130.2 ± 45.1^b^	78.13 ± 0.00^a^
GTM	*Streptococcus*	417 ± 180^a^	156.25 ± 0.0^a^	78.13 ± 0.00^a^	65.1 ± 22.6^a^	19.53 ± 0.00^a^	9.766 ± 0.000^a^
	*Klebsiella*	521 ± 180^a^	260.4 ± 90.2^a^	208.3 ± 90.2^a^	130.2 ± 45.1^a^	39.06 ± 0.00^a^	26.04 ± 11.28^a^
GAM	*Streptococcus*	521 ± 180^a^	260.4 ± 90.2^a^	130.2 ± 45.1^a^	78.13 ± 0.00^a^	39.06 ± 0.00^a^	26.04 ± 11.28^a^
	*Klebsiella*	625.0 ± 0.0^a^	312.5 ± 0.0^a^	208.3 ± 90.2^a^	130.2 ± 45.1^a^	104.2 ± 45.1^a^	78.13 ± 0.00^b^

Different superscript letter between *S. agalactiae* and *K. pneumoniae* against each preparation at each incubation hour indicate a significant difference (*p* < 0.05). GT, tylosin stabilized in sodium alginate gel; GTM, tylosin and MgO nanoparticles stabilized in sodium alginate gel; GAM, ampicillin and MgO nanoparticles stabilized in gel.

## 3. Discussion

### 3.1. Prevalence and resistance

China in a study published in year 2022 has reported 105 strains of *S. agalactiae* out of 313 clinical mastitis samples thus accounting for 33.55% prevalence on phenotypic basis of identification. The prevalence in the current study was lower than Kurjogi and Kaliwal (13), i.e., 28.10%. The pathogen is said to cause infection not only in bovines but also in humans and aquatic animals (3), so the rise in prevalence is justifiable as the pathogen spreads at one health interface. The current study showed a high prevalence of *S. agalactiae* compared with the study by Permatasari et al., (14). A study from Bangladesh reported 62.5% *Klebsiella* sp. in mastitic milk (15), which is much higher than that observed in the current study.

Previous studies conducted in the USA on *Klebsiella* spp. showed 5–19.5% resistance against the tetracycline group and up to 6.9% resistance against β-lactam. Similarly, up to 32% resistance against cefquinome, kanamycin, ceftiofur, polymyxin B, and tetracycline was found against *Klebsiella* spp (14). However, our findings reported otherwise by demonstrating the effective response of *K. pneumoniae* against penicillin, tetracycline, and amoxicillin. Contrary to the findings of our study, *S. agalactiae* showed 90% resistance against chloramphenicol, 78% against clindamycin, and 72% against enrofloxacin in previous studies, while the most sensitive response was shown against gentamicin (15).

### 3.2. Characterization of nanoparticles and their efficacy

The characterization of nanoparticles in the current study were in line with previously reported by Zaheer et al. (16). Similar findings were made in recent studies. Some studies reported smooth surfaces with a well-dispersed form, whereas others showed some nanoparticles clumped together. The average size of spherical nanoparticles ranged between 7 and 38 nm. Azam et al., (17) reported nanoparticles that ranged between 30 and 80 mm and were compactly aggregated. MgO nanoparticles have shown significant antibacterial activity, reducing growth by more than 90% at doses >5 mg/mL (18). In our study, there was a significant reduction in MIC with GAM after 8 h of incubation for each preparation, which was sustained until 16 h of incubation; this is in line with the findings from a previous study in which MgO was tested against *S. aureus* (19). Similar to our study, Aymen et al., (5) reported that MgO showed significant differences in MIC (*P* < 0.05) at the 20th hour of incubation compared with the 4th hour. According to Dehkordi et al. (20), CuO and MgO nanoparticles inhibit a wide variety of bacterial species. Siddiqi and Husen (19) reported that the antibacterial activity of ZnO nanoparticles was 1.8 ± 0.1 mg/mL. Additionally, the antibacterial activity of Fe_2_O_3_ has been shown to be 0.3125 ± 0.00 mg/mL (21, 22).

### 3.3. Efficacy of gel-based composites of antibiotics and nanoparticles

The antibacterial activity of ampicillin and florfenicol has been reported to increase when combined with silver nanoparticles against gram-positive and gram-negative bacteria. The enhancement in antimicrobial potential of antibiotics and nanoparticles stabilized in sodium alginate was also noted against bacterial pathogens (23, 24). They reported a significant reduction in the growth of bacteria with sodium alginate gel in combination with propolis. The current study was in line with the findings of (25), who reported a time-dependent increase in the antibacterial activity of sodium alginate-stabilized composites of antibiotics and MgO nanoparticles. The composites significantly reduced the MIC after incubation for 8–12 h. By forming reactive oxygen species (ROS), magnesium oxide inactivates pathogens. However, the adsorption process and direct cell membrane penetration may also be effective means of inactivating bacteria (26).

One theory for the primary mechanism of bacterial lysis is the action of ROS produced by MgO nanoparticles. This idea is reinforced by the inclusion of ascorbic acid, a ROS scavenger, in the formula (27). Therefore, it can be inferred that these ROS play a significant role in the breakdown of microorganisms and that the bacterium continues to grow and reproduce normally in their absence. According to the literature, MgO nanoparticles can control ROS formation by adjusting their size, shape, surface charge, surface area, ion release, and alkalinity (28, 29). A review on MgO nanoparticles as an antibacterial (29) reads alkaline earth metallic oxides being covered with OH^−^ layers. As MgO solution is naturally alkaline and superoxide ions are chemically stable in alkalis. The concentrated layers of superoxides may be present on the surface of the MgO powder while equilibrium of superoxide with hydroperoxyl radical (${\text{HO}}_{2}^{\cdot }$) is maintained as ${\text{O}}_{2}^{-}$ + H^+^ ← ${\text{HO}}_{2}^{\cdot }$. The ${\text{HO}}_{2}^{\cdot }$ is created when the MgO powder interacts with substances such as a bacterial cell would improve antibacterial activity. Additionally, the Mg^2+^ ions produced from the mitochondria might deactivate cellular enzymes or inhibit mitochondrial respiration, which raises the levels of ROS in the mitochondria (30). The increased ROS generation puts the cells under oxidative stress, oxidizing the components of their membranes and ultimately jeopardizing their integrity, resulting in necrosis. The mechanism underlying the ability of these nanoparticles to kill cells is not yet fully understood. To increase the antibacterial activity of nano-MgO, other polymeric additions have been used, such as chitosan (31), polylactic acid (32), or starch (33). However, the mechanism behind their synergistic effects is also not yet known. It is evident that the innovation of this work rests in its original synthesis method, its in-depth understanding of the quantity of ROS created, and the reduction of activity in the presence of a radical scavenger.

### 3.4. Toxicity analysis

The findings of the current study were in line with those of Ge et al. (26), who found that MgO nanoparticles were only slightly toxic to human cells at low concentrations (0.3 mg/mL). As a result of advanced strategies, antimicrobial activities have been improved and undesirable nanoparticle side effects have been reduced. The toxcicity of nanoparticles not only depend upon physical and chemical characteristics of nanoparticles but also on cell types being tested (34). This outcome is a result of the packaging of multiple antimicrobial agents into one nanoparticle, the coating of nanoparticles with biodegradable materials, and the engineering of target-specific nanoparticles for delivery to infection sites (35). However, further studies are required on the development of effective regimens against pathogens.

## 4. Materials and methods

### 4.1. Toxicity analysis using *Allium cepa*

For the telophase test, small onions were merged in solutions with different concentrations for 48 h. The test involved the following three groups: (1) a negative control group, in which only distilled water was used; (2) a positive control group, in which methyl methanesulfonate (MMS) (10 μg/mL) was used; and (3) a treatment group, in which different concentrations of MgO nanoparticles (M) were used. The onions were incubated in a dark room at room temperature for two different periods, 24 h and 48 h, and the concentrations used in the treatment groups were 1.25, 2.5, and 5 mg/mL. The root tips, after incubation, were fixed in 3:1 (v/v) ethanol:acetic acid, following which root tips were washed with distilled water and finally fixed in 70% ethanol. A total of eight root tips from every treatment were hydrolyzed with 1N HCl at 60°C for 10 min and then rinsed with water. Root tips were stained with Schiff's reagent at room temperature for 30 min. Apical tips were crushed on slides with acetic acid (45%) and examined under a microscope using cover slips placed on the tips. To check for mitotic activity, more than 500 cells were counted for each treatment and the mitotic index and phase index were calculated as follows:


$$\begin{array}{ccc} MI & = & \frac{Number\;of\;cells\;in\;division}{Number\;of\;total\;cells}x100\\ Phase\;index\; & = & \frac{Particular\;phase\;}{Number\;of\;cells\;in\;division}x100 \end{array}$$

#### 4.1.1. Comet assay on *A. cepa* root tips

The treated and control groups were compared using the root tips of onion bulbs. Root tips were crushed with nuclear isolation buffer (600 μL, pH 7.5) to isolate nuclei. Following centrifugation (1,200 rpm, 4°C for 7 min), the nuclear suspension was placed on slides coated with 1% normal melting point agarose at 37°C. Slides were kept on ice for 5 min, following which coverslips were removed and slides were immersed in fresh buffer in an electrophoresis tank for 20 min at 300 mA. Staining of slides was carried out for 5 min with ethidium bromide. A fluorescence microscope was used to analyze the slides (three from each sample) for DNA damage, which was classified using a 0–4 qualitative scale, with a particular focus on head integrity and tail length (36). The following formula was used to calculate DNA damage in arbitrary units:


(1) $$\begin{array}{c} Arbitrary\;Unit=\overset{4}{\underset{i=0}{\sum }}Ni\times i \end{array}$$

*Ni* = Number of cells

*i* = degree of damage (0–4).

### 4.2. The isolation and characterization of bacteria

The dairy farms located at the border of Lodhran District and Bahawalpur District consented to sample collection. A dairy farm greater than *n* = 20 animals in production was selected for sampling. Convenient sampling techniques were used to collect milk samples from 210 cattle. Milk samples presenting clinical or subclinical mastitis were collected using standard protocols. Screening for subclinical mastitis was carried out following the protocol described by Muhammad et al. (37). *S. agalactiae* and *K. pneumoniae* were identified using pooled information obtained through a series of biochemical tests described in Bergey's Manual of Determinative Bacteriology (38).

### 4.3. Antibiogram of bacteria

Biochemically characterized *S. agalactiae* and *K. pneumoniae* were tested for antibiotic susceptibility using the disc diffusion method described by Bauer et al. (39). Briefly, freshly grown bacteria (24 h) were spread over Mueller Hinton agar (10^8^ CFU/mL), upon which 10 antibiotics were placed following the guidelines of the Clinical and Laboratory Standard Institute (40). ZOI were recorded following incubation at 37°C for 24 h. Means and standard deviations were measured to assess the antibacterial potential of *S. agalactiae* and *K. pneumoniae*.

### 4.4. Preparation and characterization of magnesium oxide nanoparticles

MgO nanoparticles were prepared using the method described by Parashar et al. (41) using MgCl_2_ 0.6H_2_O, sodium dodecyl sulfate, and 2.5 M NaOH solution. The preparation protocol has been described in our previous study. XRD and SEM analysis was performed to determine the shape and dimensions of MgO nanoparticles. XRD analysis of nanoparticles was carried out using a powder diffractometer Rigaku D/max Ultima III operated at 40 kV and 0.130 A. Cu-Kα radiation was applied as a source emitting at a wavelength of 0.15406 nm, and a Quanta 250 SEM operating at 30 kV was used to obtain images of the nanoparticles (42).

### 4.5. Stabilization of MgO nanoparticles and antibiotics in sodium alginate

Sodium alginate solution (2% m/v) and gelatin solution (2% m/v) were made in water. These solutions were mixed at an 80:20 ratio (sodium alginate:gelatin) and homogenized for 2 h at 500 rpm with a mechanical stirrer to obtain sodium alginate gel (G). MgO nanoparticles (1.5 g) were added to 20 mL of sodium alginate gel and stirred for 4 h at 500 rpm to stabilize nanoparticles in the gel. Antibiotic (0.035 g) was dissolved in 20 mL of distilled water mixed with 20 mL of gel, and stirred for 4 h at 500 rpm. Then, the product was dried and ground to a fine powder. Nanoparticles and antibiotics were mixed with 20 mL of gel and stirred for 4 h at 500 rpm (23).

The following products/composites were prepared for further study: tylosin stabilized in sodium alginate gel (GT); ampicillin stabilized in sodium alginate gel (GA); MgO nanoparticles and tylosin simultaneously stabilized in sodium alginate gel (GTM); and MgO nanoparticles and ampicillin simultaneously stabilized in sodium alginate gel (GTA).

### 4.6. Resistance modulation assays

For this trial, *S. agalactiae* and *K. pneumoniae* were selected based on their minimum ZOI against more than three different classes of antibiotics. The selected isolates were used in well diffusion and broth microdilution assays to estimate resistance modulation and determine the least effective dose of gel-based preparations, respectively.

#### 4.6.1. Agar-based assay

Fresh growth of *S. agalactiae* and *K. pneumoniae* was adjusted to 10^8^ CFU/mL. Preparation of assembly for agar based was the same as described in the previous section “4.3. Antibiogram of bacteria”. The modification done included making 6–8 mm wells by well borer and followed by swabbing of bacteria. Lastly, the preparations were poured in the wells aseptically and the put to incubation for 24 hours at 37°C. Zone of inhibitions were measured following the incubation to find comparative antibacterial potential of the preparations.

Preparation of assembly for agar based was the same as described in the previous section of “antibiogram of bacteria,” except wells of 6–8 mm were made by well borer for the pouring of preparations (39).

#### 4.6.2. Broth microdilution method

The broth microdilution method was carried out to determine the MIC of gel-based preparations every 4 h during incubation. For this purpose, sterile Mueller Hinton broth (50 μL) was added to every well of a 96-well microtitration plate, following which a twofold dilution (starting from 10,000 μg/mL) of composites (sodium alginate-stabilized nanoparticles and sodium alginate-stabilized antibiotics) were added to all wells except for the positive control. Bacteria adjusted to 10^5^ CFU/mL were aseptically poured into all 96-well titration plate except the negative control well. The positive control well consisted of broth and bacteria and the negative control only consisted of broth. The optical density (OD) value was obtained at a wavelength of 695 nm using an ELISA reader at 0, 4, 8, 12, 16, 20, and 24 h of incubation. The net optical density value was calculated by subtracting the OD value at 0 h of incubation from the OD value at the different time intervals. MICs of different preparations were compared among each other, and within each preparation at different time intervals were calculated and compared similar as described in previous studies (43, 44).

### 4.7. Statistical analysis

Univariate data were analyzed through descriptive statistics and parametric tests were applied for quantitative data from two or more than two groups using a t-test and ANOVA, respectively. For comparison of means, Tukey's test was applied as a *post hoc* test. All the data were analyzed with a 5% probability using SPSS version 22.

## 5. Conclusion

The current study found an increase in the prevalence and drug resistance of *K. pneumoniae* and *S. agalactiae*. Sodium alginate-stabilized nanoparticles and antibiotics showed significant antibacterial activity against both bacteria. The preparation with antibiotics and MgO nanoparticles stabilized in sodium alginate gel exhibited a higher antibacterial response. In addition, the study concluded that stabilized preparations have the highest activity at the earliest stages of incubation, suggesting their effectiveness in outbreaks. The nanoparticles used in this study were found to be safe based on their cytotoxicity trials compared with the positive control. To overcome the difficulties associated with treating milk-borne pathogens, we should shift from the conventional worldview to the nanoworld view. A combination of non-antibiotic and antibiotic sources is a desirable candidate to replace, refine, and reduce the use of antibiotics to counter the spread of resistance. Further studies on various molecular aspects of responses of pathogens and the formulation of effective therapeutic regimens are required.

## Data availability statement

The raw data supporting the conclusions of this article will be made available by the authors, without undue reservation.

## Ethics statement

The animal study was reviewed and approved by Cholistan University of Veterinary and Animal Sciences Bahawalpur Pakistan Ethical review committee, No. ORIC No. 211. Written informed consent was obtained from the owners for the participation of their animals in this study.

## Author contributions

Conceptualization and project administration: AIA, AMn, HM, and KA. Methodology: AMn, AIA, AS, MA, and SRK. Software: KA, CM, KN, MM, AM, and AIA. Formal analysis and supervision: AIA, MS, IA, and HM. Investigation and data curation: AIA and AMn. Resources: MS, IA, and AIA. Writing—original draft preparation: AMn, MM, and AIA. Writing—review and editing: AIA, AS, IA, MS, HM, SRK, AMn, CM, and KN. All authors have read and agreed the published version of the manuscript.
